# Supplementary material for: Astrovirus Infection in Cattle with Nonsuppurative Meningoencephalitis in South Korea
Source: Viruses. 2021 Sep 28;13(10):1941. doi: 10.3390/v13101941 (PMC8537357; doi:10.3390/v13101941)
Supplement: Supplementary file 1 [file viruses-13-01941-s001.zip › viruses-1372591-supplementary/viruses-1372591-supplementary_.zip!/viruses-1372591-supplementary .pdf]

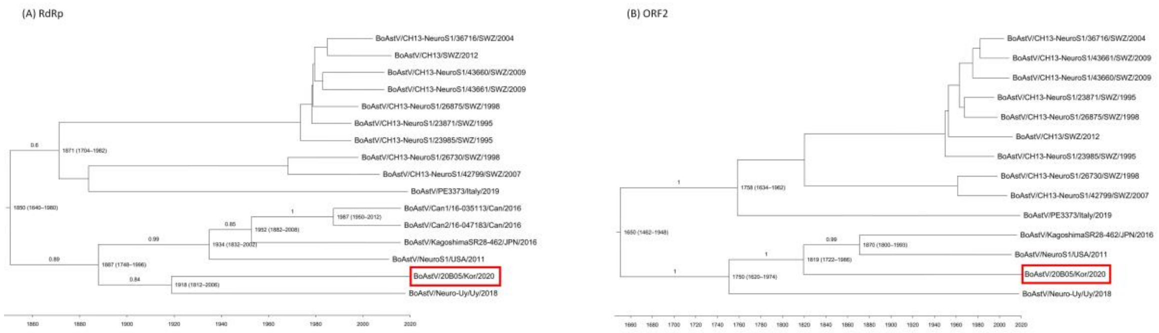

Figure S1. Maximum clade credibility trees obtained by analysis of partial RdRp and ORF2 sequences of BoAstV 20B05.
